# Materials inspired by mathematics

**DOI:** 10.1080/14686996.2016.1180233

**Published:** 2016-06-08

**Authors:** Motoko Kotani, Susumu Ikeda

**Affiliations:** ^a^ WPI-Advanced Institute for Materials Research (WPI-AIMR), Tohoku University, Sendai, Japan; ^b^ Mathematical Institute, Graduate School of Science, Tohoku University, Sendai, Japan

**Keywords:** Multiscale hierarchical materials, non equilibrium materials, carbon networks, disordered systems, data-driven materials design, topological data analysis, 60 New topics/Others, 402 Multi-scale modeling, 400 Modeling/Simulations, 404 Materials informatics/genomics, 400 Modeling/Simulations

## Abstract

Our world is transforming into an interacting system of the physical world and the digital world. What will be the materials science in the new era? With the rising expectations of the rapid development of computers, information science and mathematical science including statistics and probability theory, ‘data-driven materials design’ has become a common term. There is knowledge and experience gained in the physical world in the form of know-how and recipes for the creation of material. An important key is how we establish vocabulary and grammar to translate them into the language of the digital world. In this article, we outline how materials science develops when it encounters mathematics, showing some emerging directions.

## Graphical abstract

**Figure:**
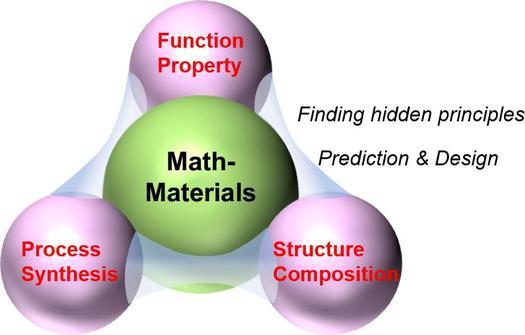

## 1. A new society in the interacting system of two worlds

Society is facing a revolution. Owing to the advance of information communication technology, the *digital world* is rapidly expanding and establishing a reality just like the *physical world* that we are familiar with. Our world is now transforming into an interacting system of these two worlds. Throughout human history, we have gained much knowledge and experience of the phenomena of the physical world. Meanwhile, we know nothing of the emerging new world – the digital world – which is governed by completely different laws. We need a new vocabulary, new grammar, and new laws for the system, and we need to develop new values and ecosystems. Mathematics has provided a vocabulary and grammar for the physical world. What roles can the mathematician play in the new era?

‘Data-driven materials design’ has become a common term. The discovery of novel materials may change our lives dramatically and provide huge benefits to society, but such discoveries are mostly accidental. Such advancement thus requires much investment in trial-and-error research and takes a long time. With the rising expectations of high-performance computers and the rapid development of information science and mathematical science (including statistics and probability theory), projects that make use of, for example, materials genomics, materials informatics, and materials integration have been established for materials development around the world. In the future, materials designs will be more systematic and employ computer-assisted approaches based on artificial intelligence for data searching and classification, but the consensus is that this may take some time. It is not enough to analyze data without *proper descriptors of the relation between structures and functions* – one should identify frameworks that can be used to predict structures with desired functions and properties by solving *inverse problems.* In other words, we have to find ways of taking into account mechanisms hidden in the complex materials system that exhibits properties and functions. There is knowledge and experience gained in the physical world in the form of know-how and recipes for the creation of materials. An important key is how we establish vocabulary and grammar to translate them into the language of the digital world. Materials science is an empirical science and largely depends on experiences accumulated by individual researchers, e.g. how we can find the optimum chemical composition, heating temperature, or pressure.

## 2. Evolution of materials science

In prehistory, people used natural materials such as stone, wood, leather, some metals, ceramics (i.e. bones and shells), and glass. With the development of metal refining technology in prehistory, the Stone Age transitioned into the Bronze Age and then the Iron Age. People now use a wide variety of materials, such as rubber, steel, silicon, glass, ceramics, cloth, paper, and wood, and materials science has emerged as a scientific field of enquiry. Materials science is an interdisciplinary field of research consisting of metallurgy, polymer science, ceramics, solid-state physics including the study of semiconductors, and many other related or subdivided fields.

In the twentieth century, modern science rapidly developed with subdivision of research fields. It is evident that the subdivision of research fields helped deepen the understanding of each field and enhanced the development of science. Meanwhile, the height of barriers separating subdivided fields gradually increased and different vocabularies and terminologies appeared even in related fields. In the field of physics, it is often said that Arnold J. Sommerfeld (1868–1951) was the last physicist who could explain all physics.[1] This statement is valid: in the latter part of the twentieth century, no single person completely understood all of physics. This situation also occurred in materials science. Scientists developed techniques for the synthesis of new organic and inorganic materials and the spectrum of materials science rapidly expanded. Much effort is required to understand the details of different fields of materials science. Interdisciplinary interaction is an attempt to reintegrate such subdivided fields. Today, in every scientific field, it is often said that interdisciplinary integration leads us to the next innovation. However, it is not clear how interdisciplinary integration leads to new findings, as there are few successful examples. Here we would like to share an example that awaits a comprehensive explanation through interdisciplinary research.

**Figure 1.**
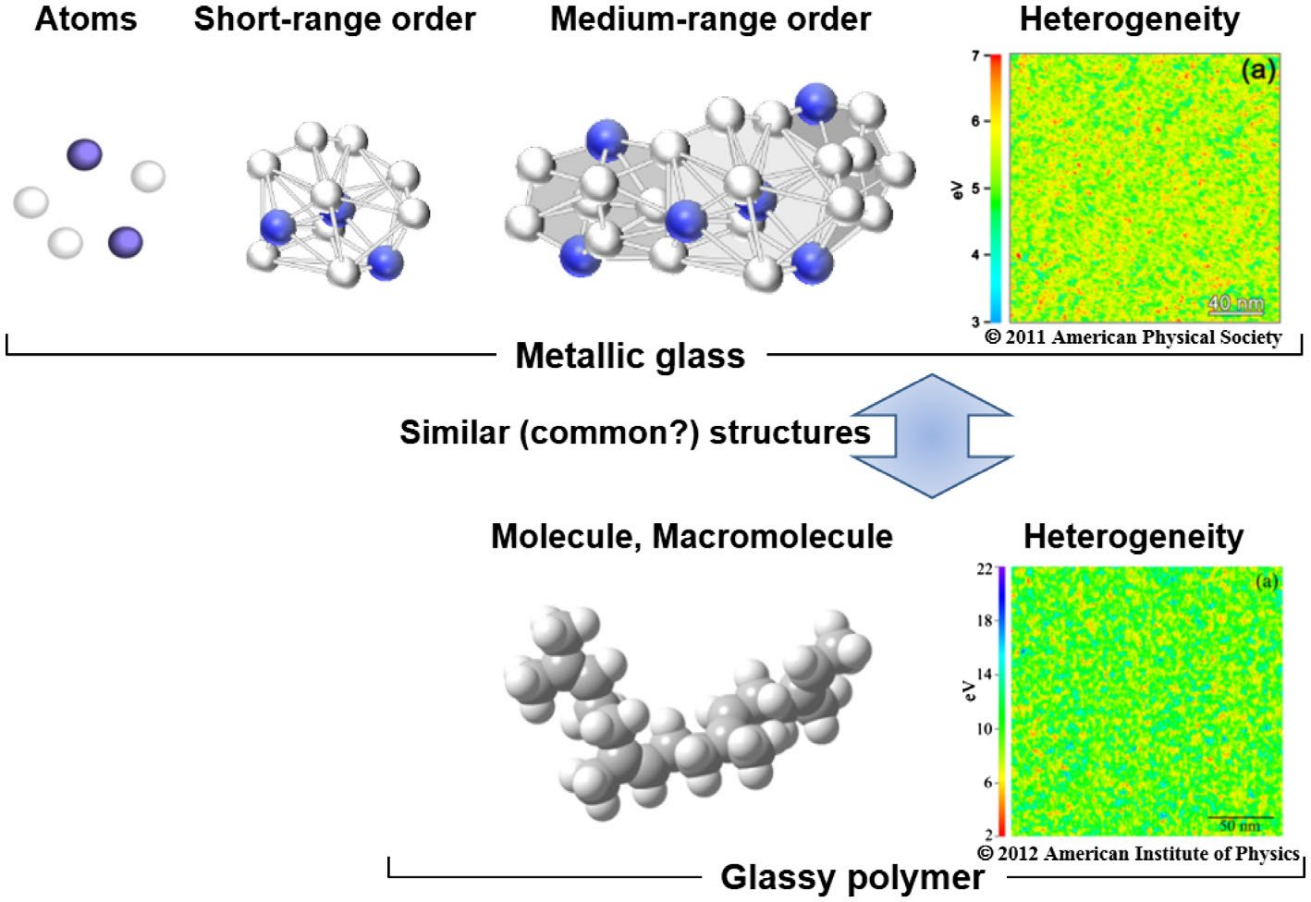
Schematic chart showing the similarity between metallic glass and glassy polymer in molecules and nanostructures. Energy dissipation maps are reproduced from [4] and [5] with permission of American Physical Society and American Institute of Physics, respectively.

The shear transformation zone (STZ) is an important concept relating to the mechanism of plastic deformation of metallic glass which has no dislocation movement. The STZ is a zone of nanoscale volume that plastically flows when stress is applied. It is thought that the concentration of stresses to a local STZ creates a shear zone, which leads to mechanical destruction and low ductility. Our colleague, Prof. Mingwei Chen, studying metallic glasses developed a ew experimental method that evaluates the STZ, and revealed its size to be about 2 nm [2]. Another colleague, Prof. Ken Nakajima, studying polymers, developed a new technique that maps the local viscosity distribution on a nanometer scale using atomic force microscopy [3], and applied it to investigate the heterogeneity in metallic glasses in collaboration with Chen’s group. The analysis revealed that the inhomogeneous structure of the viscosity measured in metallic glasses had a distinctive scale of 2.5 nm, which matches that of STZs [4]; this result strongly suggests that the inhomogeneous distribution of viscosity and STZs are strongly related. The most interesting point is that this distinctive size is almost the same as a cooperatively rearranging region (CRR) observed in glassy polymers.[5] Although further investigation is required, we suspect that a common mechanism lies beneath both metallic glass and glassy polymer, as shown in Figure 1. An important step in gaining a comprehensive understanding of glassy materials is to find a universal framework that can be used to describe the two phenomena. Once we have a translator between two different systems, ideas of one system can be translated to another.

## 3. Mathematics in history: continuum versus discrete

‘The grand book of the universe is written in the language of mathematics’, said Galileo Galilei, an Italian astronomer, physicist, engineer, philosopher, and mathematician. Mathematics has provided the common language of science and technology since ancient times. Calculus (differentiation and integration) founded by Isaac Newton and Gottfried Leibniz independently in the seventeenth century is a particularly powerful tool used to describe physical phenomena in terms of differential equations. It is not only used to analyze shapes of objects but also motions according to the principle of least action*.* This enables us to predict deterministic physical phenomena. Pierre de Fermat and Blaise Pascal introduced again in the seventeenth century the concept of probability, and Andrey Kolmogorov in the twentieth century founded modern probability theory, which provides rigorous treatment of uncertainties. As a consequence, predicting random phenomena is possible using stochastic differential equations. It also gives a logical basis of quantum mechanics. The usefulness of differential equations based on mathematical modeling and numerical analysis has been recognized, as in Eugene Wigner’s article ‘The unreasonable effectiveness of mathematics in the natural science’ published in 1960 [6]*.* However, differential equations do not work in a digital or discrete world (i.e. for microscopic features).

Before the digital world emerged in cyber space, materials science encountered the digital (discrete) world. We developed technology to observe and control atoms and molecules whose sizes and rules were beyond our experience and intuition and encountered complex systems where two worlds, the digital and the physical, interacted with each other. That is to say, we considered materials as *systems of hierarchical networks*, where microscopic structures are discrete (digital data) and macroscopic properties are continuous (physical phenomena), and recognized the importance of multiscale analysis in establishing a *multiphysical system* that bridges layers of the hierarchical network, or in other words, *connects the discrete (digital) and continuous (physical) worlds*.

**Figure 2.**
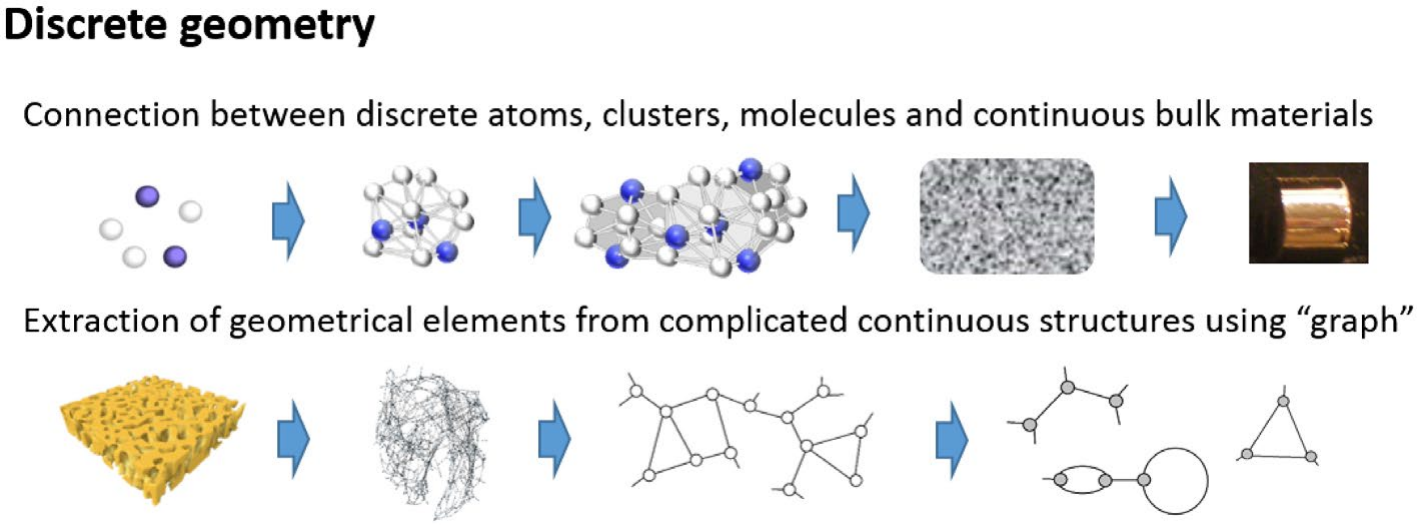
Examples showing the power of geometry. Connection of continuum and discrete elements in materials, and extraction of discrete geometrical elements.

There has been increased demand outside of the mathematics community for mathematical theories and tools that can describe the discrete world and thus the relations between discrete and continuous worlds. Coincidentally, toward the end of the twentieth century, mathematics was expanded to the discrete world, such as in the cases of discrete group theory (geometric group theory), discrete geometry, and discrete geometric analysis, with an interest in deepening mathematical theories. Mathematics is ready to offer new tools that will bridge the digital world and physical world, or to understand relations between layers of hierarchical systems, as shown in Figure 2.

## 4. Mathematics for materials

In this section examples are given of mathematics used in the study of matter and materials and some emerging collaborations are introduced.

### 4.1. Orders hidden in disordered systems

The first interaction between mathematics and materials was possibly seen in the concept of atomism in ancient Greece.[7] Atomism was forgotten until modern atomism was constructed by John Dalton in the early 1800s. At the end of the nineteenth century and in the twentieth century, crystallography for the study of atomic arrangements in crystals advanced based on the theoretical discovery of 230 space groups and experimental discovery of X-ray diffraction. A mathematical model of atomic arrangements is given by a graph. A crystal lattices is defined as an abelian covering of a finite graph in a mathematical terminology and gives a toy model of the atomic arrangement of crystals.[8, 9] Here, group theory is used to describe symmetries and periodicities in the atomic arrangements. When the quasicrystal was discovered,[10] many people believed there must be hidden symmetries or orders, although there is no periodic structure observed in the physical world. Advanced mathematics provides the vocabulary of noncommutative geometry,[11] an advanced notion of an abelian (commutative) group, for an aperiodic system like a quasicrystal,[12] to identify orders hidden in disordered systems (Figure 2). In the framework, one can generalize methods developed for crystals and apply them to aperiodic systems.

### 4.2. Topological functional materials

It is essential to understand relations between structures and properties/functions of materials. Although we have plenty of data of complex structures of materials, we cannot study such structures in a systematic way because there are few geometric descriptors known to materials science. There are many useful concepts available, such as differential geometry for macroscopic structures and discrete differential geometry for atomic networks and nanostructures.

Topology is a mathematical concept for describing a shape up to continuous deformation to abstract essential geometric properties from a complex shape. It is therefore used to describe complex structures that are robust under environmental change but have highly sensitive properties at the same time.

Topology was first introduced by Leonhard Euler, and Enrico Betti and Henri Poincare established the basis of algebraic topology to figure topological invariants. Homology is a notion in algebraic topology developed in the twentieth century to count loops and holes and to study their connectivity. Topological data analysis, *persistent homology* introduced by Herbert Edelsbrunner and his colleagues in 2002 [13] in particular, is useful for identifying hidden orders in amorphous materials, polymers, or composite materials through collaborations at the Advanced Institute for Materials Research.[14–16]

Quantum materials are materials with special features resulting from the quantum behavior of electrons. They are studied according to the band structure of Schrödinger operators. Topologically protected surface states were theoretically predicted by Kane and Mele in 2005,[17] and subsequently confirmed by many similar predictions for different systems and experiences. A mathematical framework for the comprehensive understanding of these phenomena is expected to be developed and extended to more general settings. The index theorem for a noncommutative geometry or coarse geometry is now used.[18–22]

### 4.3. Discrete differential geometry for carbon networks

**Figure 3.**
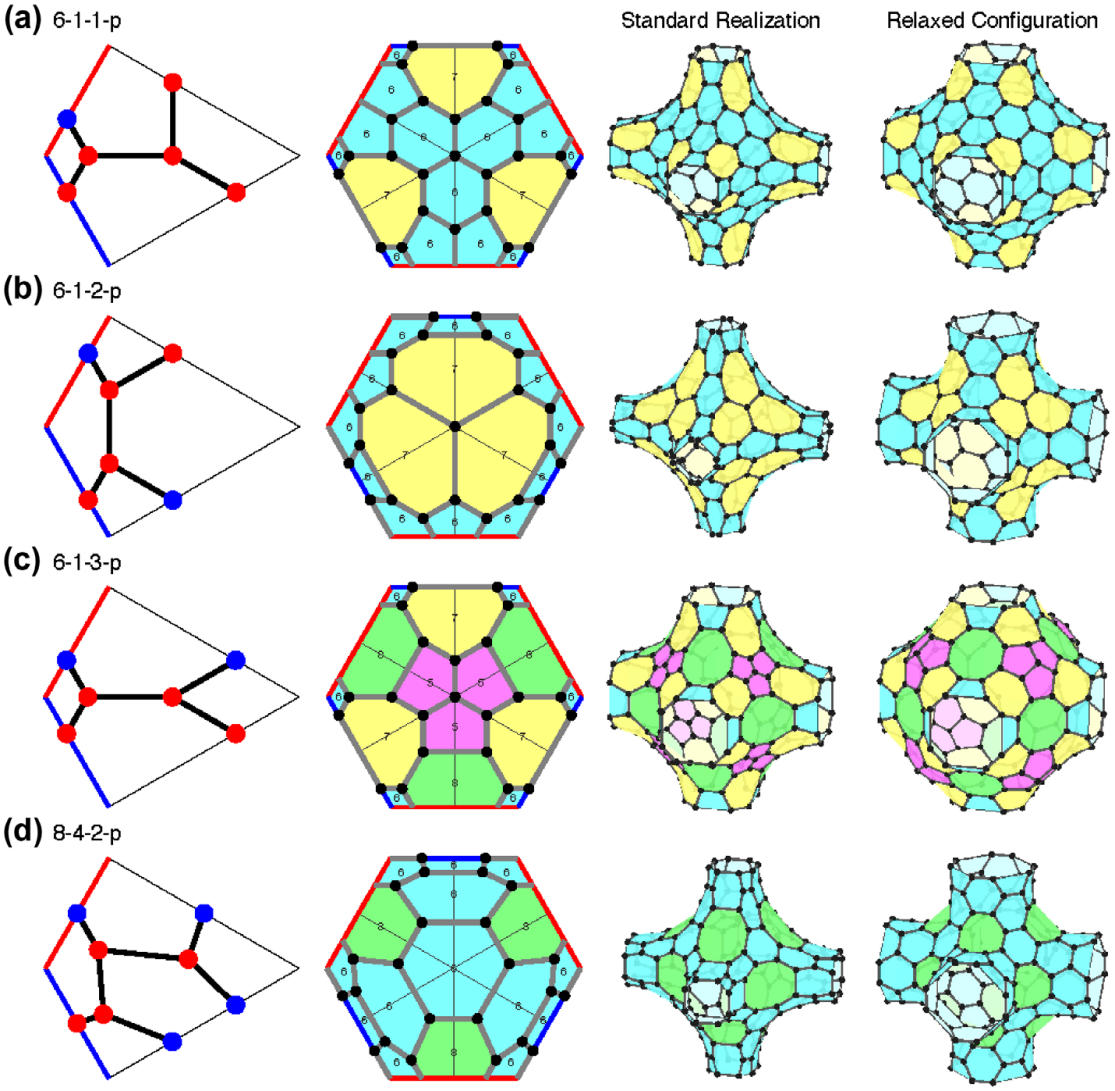
Mackay-like crystals. Reprinted from [25]. Copyright (2014), with permission from Elsevier.

In 2008, the mathematician Toshikazu Sunada wrote an article ‘Crystals that nature might miss creating’.[23] He classified three-dimensional networks that had some symmetry and strong isotropy and found that there were two classification groups: one is the atomic structure of diamond, and the other is the diamond twin, which he did not know and called the K_4_ lattice. The carbon K_4_ lattice, if it exists, is a metal according to first-principle calculation.[24] It turned out later that the structure has been rediscovered many times in history, in the long list of the classification by crystallographers such as the silicon network in SrSi_2_. Yet it remains important that Sunada showed the beauty of the structure from a mathematical viewpoint and that he challenged materials scientists to synthesize it. There are many existing carbon networks, such as diamond, fullerenes, and carbon nanotubes, that are natural and beautiful from a mathematical viewpoint. Meanwhile, there are many new structures proposed or synthesized by organic chemists for which there are no descriptions or explanations. It seems that this is a domain in which we can expect fruitful collaborations between materials scientists and mathematicians. Recently, we began investigating carbon networks with negative curvatures – Mackay-like crystals – by applying notions in differential geometry [25] as shown in Figure 3. Among them, there is a structure with an interesting electrical property called a Wyle semimetal.[26] Collaboration also inspired mathematicians to develop a discrete version of differential geometry, a new branch of mathematics.[27]

### 4.4. Multiscale hierarchical materials based on discrete geometric analysis

Innovative functional materials can be created only by recognizing the complex multiscale hierarchical structure in materials systems from the atom/molecule scale to the macroscopic scale of materials and devices. Precise structure analysis and control at each level of hierarchy from the atom/molecule scale will be carried out using advanced equipment and new technology. Discrete geometric analysis provides a bridge between scales and takes account of detailed geometric data. By employing these advanced tools, we are attempting to produce functional multiscale hierarchical materials.

The graph is an important and useful tool in discrete geometric analysis. Nanoporous materials are attracting much interest owing to their promising applications in catalysts and capacitors. The three-dimensional (3D) network structure of nanopores is directly related to the physical and chemical properties of nanoporous materials and it is important how the 3D structure is analyzed. The use of graph theory is a powerful way of investigating such 3D network structures and it can derive important parameters, for example, related to material transport through complicated nanopore structures.[28]

### 4.5. Nonequilibrium materials based on mathematical dynamical systems

One of the major challenges in materials science is to synthesize multifunctional materials, where multiple functions emerge according to nonequilibrium states, hybrid structures consisting of different types of materials, or the inhomogeneity of systems. On the basis of a mathematical dynamical system, we will focus on clarifying mechanisms of dynamical structural formulation in nonequilibrium systems. This will enable us to accurately control nonequilibrium and inhomogeneous materials and to achieve prescribed multiple functions for a given environment.

Dynamical systems are often accompanied by stochastic processes and treatment of the stochastic processes. For example, in metallic glasses, shear bands appear when continuous stress is applied but the timing of the appearance is almost random and it is difficult to predict or control it. This is a problem for the application of stochastic theory and some results have been reported.[29]

## 5. Recent direction of mathematical science

Although mathematics has advanced by interacting with other scientific fields throughout history, in the latter half of the twentieth century mathematicians concentrated on reconstructing its modern framework and deepening abstract theories motivated by their own interests, and neglected the relations with other fields. It may be necessary for any discipline to have such a period of complete isolation. However, after reaching a satisfactory level of advancement, the field should open its doors to seek inspiration.

In the 1970s, Freeman Dyson lamented the deep alienation between theoretical physicists and mathematicians. In 1980, a renaissance began in the exchange between pure mathematics and theoretical physics. Toward the end of the last century, a movement emerged to promote interactions between mathematics and scientists as well as industry. In 1998, the *Odom Report* (US National Science Foundation, NSF) [30] triggered interaction, and it was followed by the *Brown Report* (US Department of Energy).[31] Following these reports, projects were established to encourage collaborations between mathematics and other scientific fields; e.g. the Multiscale Mathematics Initiative from 2004 to 2006 and Science at the Triple Point between Mathematics, Mechanics and Material Science (PIRE, NSF) from 2011 to 2015. Additionally, the Organisation for Economic Co-operation and Development published the *Report on Mathematics in Industry* in 2008[32]. They organized a Global Science Forum to discuss mechanisms for strengthening the connection between mathematics and industry. In Japan, a promotion of mathematical science started in 2006. The first JST PRESTO/CREST program in the area of mathematics was established in 2007/2008.

When breakthroughs in interdisciplinary research are expected and emerging results are seen, constant support and encouragement should be provided. Individual effort is not enough to surmount the energy barrier for the fusion reaction required to create new disciplines.

**Figure 4.**
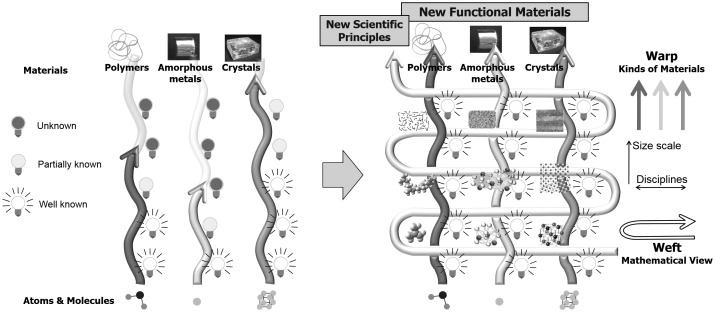
The role of mathematics presented as wefts in textiles. Modified from the literature.[33]

The authors believe that it is a good time for mathematics and materials science to encounter each other and interact. Mathematics will play the role of wefts in textiles of materials science and lead us to a comprehensive understanding beyond the barriers dividing disciplines and discontinuities existing in hierarchy from micro to macro (Figure 4). Further discussion can be found in the literature.[33]

## 6. Conclusions

Materials science has evolved with a subdivision of research fields: metallurgy, polymer science, ceramics, solid-state physics including the study of semiconductors, and many other related or subdivided fields. However, such subdivision resulted in a situation where nobody was able to comprehensively detect mutual relations and common principles existing between subdivisions. It is now entering a new stage of development. Interdisciplinary integration of materials research enables us to find common mechanisms hidden in the complex materials system that manifest properties and functions. Mathematics is ready to offer new tools to understand materials as complex hierarchical systems. Fruitful collaboration is now at hand.

## Disclosure statement

No potential conflict of interest was reported by the authors.

## Funding

This work was supported by MEXT, Japan [grant number World Premier International Research Center Initia]

## References

[CIT0001] Heisenberg WK (1971). Der Teil Und Das Ganze.

[CIT0002] Pan D, Inoue A, Sakurai T (2008). Experimental characterization of shear transformation zones for plastic flow of bulk metallic glasses. Proc Natl Acad Sci USA.

[CIT0003] Wang D, Fujinami S, Liu H (2010). Investigation of true surface morphology and nanomechanical properties of Poly(styrene- b -ethylene- co -butylene- b -styrene) using nanomechanical mapping: effects of composition. Macromolecules.

[CIT0004] Liu YH, Wang D, Nakajima K (2011). Characterization of nanoscale mechanical heterogeneity in a metallic glass by dynamic force microscopy. Phys Rev Lett.

[CIT0005] Wang D, Liu Y, Nishi T (2012). Length scale of mechanical heterogeneity in a glassy polymer determined by atomic force microscopy Appl. Phys. Lett.

[CIT0006] Wigner EP The unreasonable effectiveness of mathematics in the natural sciences. Comm. Pure Appl. Math.

[CIT0007] Cromwell PR (1997). Polyhedra: one of the most charming chapters of geometry.

[CIT0008] Kotani M, Sunada T (2003). Spectral geometry of crystal lattices. Contemp. Math.

[CIT0009] Kotani M, Sunada T (2000). Standard realization of crystal lattice via harmonic maps. Trans Amer Math Soc.

[CIT0010] Shechtman D, Blech I, Gratias D (1984). Metallic phase with long-range orientational order and no translational symmetry. Phys Rev Lett.

[CIT0011] Connes A (1994). Noncommutative geometry.

[CIT0012] Baake M, Moody RV (2000). Directions in mathematical quasicrystals: CRM monograph series.

[CIT0013] Edelsbrunner H, Cohen-Steiner D, Harer J (2002). Topological persistence and simplification. Discrete Comput Geom.

[CIT0014] Hirata A, Kang L. J, Fujita T (2013). Geometric frustration of icosahedron in metallic glasses. Science.

[CIT0015] Nakamura T, Hiraoka Y, Hirata A Description of medium-range order in amorphous structures by persistent homology.

[CIT0016] Nakamura T, Hiraoka Y, Hirata A (2015). Persistent homology and many-body atomic structure for medium-range order in the glass. Nanotechnology.

[CIT0017] Kane CL, Mele EJ (2005). Quantum spin Hall effect in graphene. Phys Rev Lett.

[CIT0018] Bellissard J, van Elst A, Schulz-Baldes H (1994). The non-commutative geometry of the quantum hall effect. J Math Phys.

[CIT0019] Schulz-Baldes H (2013). Persistence of spin edge currents in disordered quantum spin hall systems. Comm Math Phys.

[CIT0020] Kotani M, Schulz-Baldes H (2014). Villegas-Blas, Carlos quantization of interface currents. J Math Phys.

[CIT0021] Graf GM, Porta M (2013). Bulk-Edge correspondence for two-dimensional topological insulators. Comm Math Phys.

[CIT0022] Kubota Y Controlled topological phases and bulk-edge correspondence.

[CIT0023] Sunada T (2008). Crystals that nature might miss creating 2008 *Not*. Amer Math Soc.

[CIT0024] Itoh M, Kotani M, Naito H (2009). New metallic carbon crystal. Phys Rev Lett.

[CIT0025] Tagami M, Liang Y, Naito H (2014). Negatively curved cubic carbon crystals with octahedral symmetry. Carbon.

[CIT0026] Weng H, Liang Y., Xu Q. (2015). Topological node-line semimetal in three dimensional graphene network. Phys Rev B.

[CIT0027] Kotani M, Naito H, Omori T A discrete surface theory.

[CIT0028] Packwood DM, Jin T, Fujita T (2014). Mixing time of molecules inside of nanoporous gold. SIAM J Appl Math.

[CIT0029] Louzguine-Luzgin DV, Packwood D M, Xie G (2013). On deformation behavior of a Ni-based bulk metallic glass produced by flux treatment. J alloys compd.

[CIT0030] Odom W (1998). Report of the senior assessment panel for the international assessment of the U.S. mathematicalsciences. National Science Foundation (NSF).

[CIT0031] Brown DL, Bell J, Estep D (2008). Applied mathematics at the U.S. department of energy: past, present and view to the future. U.S. Department of Energy (DOE).

[CIT0032] Global Science Forum. Report on mathematics in industry. (2008). Organisation for Economic Co-operation and Development (OECD). http://www.oecd.org/science/sci-tech/41019441.pdf.

[CIT0033] Ikeda S, Kotani M (2015). A new direction in mathematics for materials science. SpringerBriefs in the mathematics of materials.

